# Risk factors for non-specific neck pain in young adults. A systematic review

**DOI:** 10.1186/s12891-020-03379-y

**Published:** 2020-06-09

**Authors:** Henriette Jahre, Margreth Grotle, Kaja Smedbråten, Kate M. Dunn, Britt Elin Øiestad

**Affiliations:** 1 Department of Physiotherapy, Oslo Metropolitan University, Postboks 4 St. Olavs plass, 0130 Oslo, Norway; 2 Research and communication unit for musculoskeletal health (FORMI), Clinic for Surgery and Neurology, Oslo University Hospital, Oslo, Norway; 3 Primary Care Centre Versus Arthritis, School of Primary, Community and Social Care, Keele University, Keele, UK

**Keywords:** Neck pain, Risk factors, Young adult, Systematic review

## Abstract

**Background:**

Young adulthood is a sensitive period of life where development of musculoskeletal neck pain may be established and impact future health. The objective of this systematic review was to investigate risk factors for non-specific neck pain in young adults.

**Methods:**

Systematic searches were conducted in six databases in September 2019. Prospective cohorts and registry studies including participants in whom the risk factor or the outcome (neck pain) was registered in the ages 18–29 years old were included. The Quality in Prognosis Studies tool was used for quality assessment. A modification of the Grading of Recommendations Assessments, Development and Evaluation was used to assess the overall quality of the evidence. Potential risk factors investigated in more than one study were summarised.

**Results:**

Searches yielded 4527 articles, of which six matched the eligibility criteria. Fifty-six potential risk factors were investigated in the six studies, covering a broad range of domains. Five risk factors were investigated in more than one study (female sex, body mass index (BMI), physical activity, duration of computer use and perceived stress). Physical activity and BMI showed no association with neck pain, and inconsistent results were found for female sex, duration of daily computer use and perceived stress. Risk of bias was moderate or high in all studies, and the overall quality of evidence was very low.

**Conclusion:**

The studies included many potential risk factors, but none of them showed consistent associations with neck pain. There is a paucity of high-quality studies investigating risk factors for neck pain in young adults.

## Background

Neck pain is one of the most common musculoskeletal disorders worldwide, with a reported 12-month prevalence ranging from 42 to 67% in young adults [1–3]. According to the Global Burden of Disease (GBD) Study, low back and neck pain were the second leading causes of years lived with disability (YLD) for young adults aged 20–24 years [4]. Furthermore, data from the GBD Study shows that neck pain is a rising problem, with a 21% increase in the population prevalence of pain lasting more than 3 months between 2006 and 2016 [5]. Neck pain is associated with disability and reduced quality of life [6], and in young adults, neck pain has been shown to be a risk factor for reduced general work productivity [7]. The economic consequences of neck pain are significant for both the individual and the society due to costs related to healthcare, insurances, loss of productivity, and sick leave [5, 6].

Young adulthood, often referred to as the age span between 18 and 29 years, is the transitional stage between adolescence and adulthood, when people are in the process of forming an adult identity [8]. This period is characterised by extensive changes, handling choices and opportunities such as moving out from home, choice of education and career, and establishing an adult lifestyle [8, 9]. Inequalities in own socioeconomic status emerge [10], and biological parameters such as bone mass [11] and muscle strength [12] peak during this period. Previous studies have shown that exposures and choices made during young adulthood influence health and well-being [13–15], therefore, we believe that this period also is vulnerable for future musculoskeletal health, such as neck pain. Furthermore, in young adulthood, one has the opportunity of changing habits from earlier life. Consequently, young adulthood may be a critical time in the life course in which the long-term development and management of musculoskeletal pain could be influenced, ideally reducing episodes of neck pain and its’ consequences in adulthood [16].

Despite the high prevalence of neck pain leading to disability, neck pain in young adulthood has attracted little attention in the literature. Previous systematic reviews have investigated risk factors for non-specific neck pain in children, adolescents and adults, but not in the stage of young adulthood [17–19]. Female sex, older age, being an ex-smoker, present or previous history of low back pain, previous episode of neck pain or psychosocial factors have been shown to be risk factors for neck pain in adults [20, 21]. Depression, mental distress and psychosomatic complaints seem to be associated with neck pain in children and adolescents [17], and daytime tiredness seems to be a risk factor for neck pain in adolescent girls [19]. It is unclear if risk factors in young adulthood reflect these findings since young adulthood differs from childhood and adolescence regarding behavioural changes, the fulfilment of social milestones, and the adjustment to contextual life changes that occur in this transitional stage of life [8]*.* The concerns are that important developmental milestones resulting in life-long individual and societal consequences are not reached. To reinforce the concepts of sensitive periods for intervention, investigation of risk factors in young adults are necessary in a life course perspective. Importantly, evidence suggests that pain, disabilities and health behaviour at younger ages tend to persist into adulthood [22–24] and that the prevalence of musculoskeletal consultations is still increasing up to the early twenties [25]. By identifying risk factors associated with the development of neck pain in young adults, new prevention strategies can be developed to minimise this prevalent and costly health problem.

The aim of this systematic review was to investigate risk factors for non-specific neck pain in young adults.

## Methods

This systematic review is reported according to the Preferred Reporting Items for Systematic Reviews and Meta-Analyses (PRISMA) statement [26] (Additional file 1). The study protocol is published in the PROSPERO database (CRD42019125008).

### Search strategy

The search strategy is described in Additional file 2. The searches were built using a variety of subject headings, keywords and synonyms for ‘young adult’ (18–29 years), ‘risk factor’, ‘cohort study’ and ‘self-reported neck pain’. The first author (HJ) performed the searches with guidance from librarians at Oslo Metropolitan University. The electronic searches were conducted with no restriction on publication date up to September 2019 in the following databases: MEDLINE, EMBASE, AMED, SPORTSDiscus, PSYCHINFO, Web of Science and CINAHL. Filters for human subjects and English or Scandinavian language were applied to the searches. Hand searches were conducted in the following journals: Spine, Pain, European Journal of Pain, BMC Musculoskeletal disorders and the Global Spine Journal. All identified publications were imported into EndNote library (Clarivate Analytics, Philadelphia, USA) where duplicates were removed. Two authors (HJ and KS) hand-searched reference lists of included studies, and reference lists of previous relevant systematic reviews.

### Eligibility criteria

Prospective cohort studies or registry studies with a follow-up period of at least 6 months were included. Participants were required to be between 18 and 29 years of age (either at time of risk factor measurement or outcome measurement) and being pain-free at the risk factor assessment (baseline). Studies involving participants that might have experienced a previous episode of neck pain were included, as long as participants were pain-free at baseline. Only results from individuals who were pain-free at baseline were included if a study included participants both with and without neck pain at that assessment. Studies that only included people with specific underlying pathology such as tumours, fractures, infection, inflammatory disorders or osteoporosis were excluded. Full-length articles in English or Scandinavian languages were included.

### Definition of outcome

The main outcome of this systematic review is self-reported, non-specific neck pain with no restrictions on pain duration or intensity. The anatomical region of the neck is as proposed from the Neck Pain Task Force as *pain from the cervical spine, and muscles and soft tissues in the cervical area with or without affecting the head, trunk or shoulders* [27].

### Risk factors

In order to distinguish between different types of potential risk factors, they were classified according to the International Classification of Functioning, Disability and Health (ICF) components into 1) body functions and structures, 2) activities and participation, 3) environmental, and 4) personal factors [28]. Potential risk factors investigated in more than one study or showed statistical significance in one study were summarised in a narrative synthesis. To be defined as a risk factor, variables had to be statistically significantly associated with a greater risk of neck pain in either adjusted or unadjusted analysis presented with a *p*-value of less than 0.05 or 95% confidence interval (CI) that did not cross 1.0. Factors with a reduced risk of neck pain (negative association) were categorised as protective factors. Because of heterogeneity between studies regarding definition of neck pain, follow-up periods and risk factors assessed, meta-analyses were not conducted.

### Study selection

Two of the review authors (HJ and KS) screened the titles and abstracts using Rayyan [29]. Full-text reports were obtained for all titles and abstracts that met the inclusion criteria. The same review authors then screened the full-text reports and made the final inclusion and exclusion of studies. Disagreements were solved by consensus between the two authors and by a third co-author (BEØ) if necessary.

### Data extraction

Two authors (HJ and BEØ) independently extracted data from all the included studies using a prepared excel (2016) sheet (Microsoft, Washington, USA) with publication details (author, year, and study site), number of subjects (participation rate and drop-out rate), study population (age, sex and neck pain status), follow-up period, outcomes, reported risk factors and statistical analysis. The sheet was piloted using two included studies to ensure that all the relevant information was extracted.

### Quality assessment

Two authors (HJ and BEØ) separately evaluated the included studies for quality using the Quality in Prognosis Studies (QUIPS) assessment tool [30]. This tool evaluates quality in prognostic studies in six domains: study participation, study attrition, prognostic/risk factor measurement, outcome measurement, study confounding and statistical analysis and reporting. A score of low, moderate or high risk of bias was assigned to each domain for each study. A total score for each study was not conducted, as suggested by the Cochrane group [31]. After individual assessment, the two authors had a consensus meeting, where disagreements were resolved by discussion.

### Assessing quality of the evidence

The quality of the evidence was conducted using a modification of the Grading of Recommendations Assessments, Development and Evaluation (GRADE) framework [32], adapted for prognosis research [33]. The quality was rated as high, moderate, low or very low. The quality of the evidence was downgraded based on early phase of investigation, study limitations, inconsistency, indirectness, imprecision and publication bias. The evidence could be upgraded based on large effect sizes and exposure-response gradients [33]. The overall quality of evidence was only rated for potential risk factors that were measured in more than one study or showed statistical significance in one study. Two review authors (HJ and BEØ) independently assessed the quality of evidence and then had a consensus meeting.

## Results

### Results from search

**Fig. 1:**
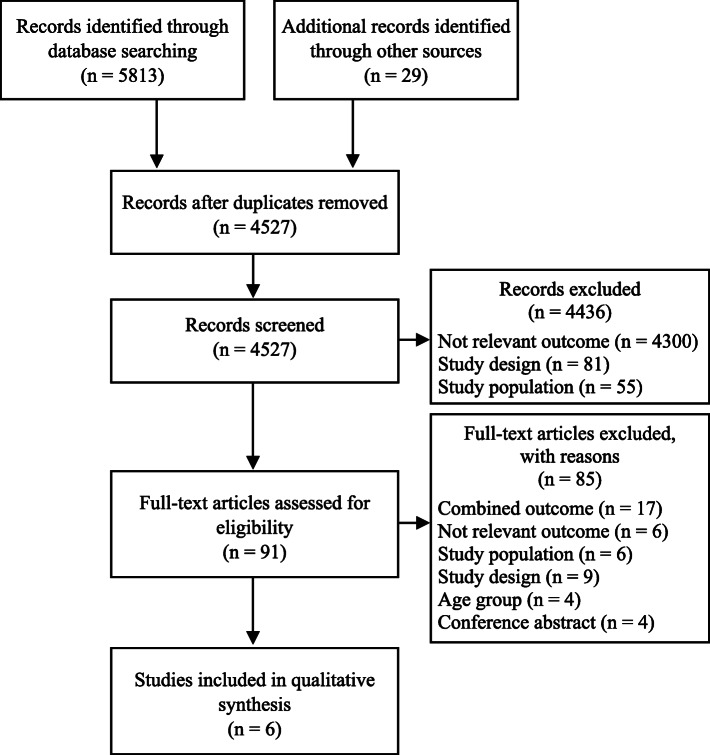
PRISMA Flowchart. Combined outcome: studies that combine neck pain with other musculoskeletal outcomes; Not relevant outcome; studies have not measured neck pain as an outcome; Study population: included participants with pain at baseline; Study design: not prospective cohort or registry studies; Age group: studies that did not include separate analysis for young adults (18–29 years); Conference abstracts: only conference abstract existed

The overall searches yielded 4527 articles after removing duplicates. Most of these articles were identified in electronic searches, with the exception of 29 articles that were found by hand searches. After screening for eligibility, six studies [34–39] were included in the final summary. A flowchart of the study selection is presented in Fig. 1. The main reasons for excluding articles were that the studies did not measure neck pain as an outcome, did not report results separately for young adults, or they did not report results separately for neck pain. A complete overview of study exclusion at the full-text stage is given in Additional file 3.

### Study characteristics

Four of the included studies were prospective cohort studies from Sweden [36], United States [37], Thailand [34] and Finland [39], and two were registry studies from Sweden [35, 38]. A total of 8856 study participants were included. Three studies [34, 36, 39] were empirical studies in a hypothesis-generating phase of the investigation, and three studies [35, 37, 38] were explanatory. The study populations consisted of university students [34, 36, 37], high school students [39], young adults from the general population [35], and young men who had performed mandatory conscription [38]. Age at baseline varied from 15 to 18 years, and follow-up time ranged from 1 to 25 years (Table 1). One study reported risk factors for first-episode of neck pain [37], and five studies reported risk factors for a combination of participants with first episodes and recurrent episodes [34–36, 38, 39].

**Table 1:** Study characteristics in alphabetical order

Authors	Population source and eligible (n)	Participants at baseline (n)	Participants at follow-up, n (% of included)	Age at baseline (mean or range)	Years of follow-up	Definition of outcome
**Dieck et al. (1985)** [37]	Female college students from an eastern U.S. College graduating in 1957–1959. Population: 1948	1713	903 (52.7%)	18–19 years	25	First episode of neck pain. Neck pain lasting for at least a week.
**Grimby-Ekmann et al. (2009)** [36]	Swedish medical and IT students from six colleges in five cities of Sweden. Invited: 1728	1204 respondents at baseline. Not reported how many included in the risk analysis at baseline.	267 (22.2%) - 326 (27%), variations because of incomplete data	18–25 years	1 and 2 years	A combination of first and recurrent episodes of neck pain. Pain at present, period of pain and the number of years with pain
**Gustafsson et al. (2017)** [35]	Swedish young adults randomly selected from the registry of the general population kept by the Swedish Tax Agency. Assessed for eligible: 20000	4431 eligible for risk analysis	1 year: 1542 (34.8%) crude analysis 1522 (34.3%) multiple analysis 5 year: 870 (19.6%) crude analysis 868 (19.6%) multiple analysis	20–24 years Mean: 22 years	1 and 5 years	A combination of first and recurrent episodes of neck pain. Currently experienced pain in the upper part of the back/neck
**Kanchanomai et al. (2011)** [34]	Undergraduate students at Thammasat University Assessed for eligibility: 3545 Eligible: 2511	684 agreed to participate in the study	524 (77%)	18–25 years Mean: 19.4 years	1 year, every 3rd month.	A combination of first and recurrent episodes of neck pain. Neck pain lasting > 24 h during the past 3 months. Neck pain for 2 or more follow-ups in a row was categorized as persistent neck pain.
**Siivola et al. (2004)** [39]	Finnish high school students. Random sample: 826	189 eligible for risk analysis	104 (55%) without neck pain at baseline	15–18 years	7 years	A combination of first and recurrent episodes of neck pain. Weekly neck and shoulder pain during the last 6 months.
**Timpka et al. (2013)** [38]	Swedish men that had performed mandatory conscription and been included in the Swedish Living Conditions Surveys. Eligible: 7123 accepted participation in survey	5489	5489	17–19 years Mean: 18.2 years	17.2 years	A combination of first and recurrent episodes of neck pain. Currently neck or shoulder pain.

Some studies [35, 36, 39] included both participants with and without pain at baseline, but they did separate risk analysis for those without pain at baseline

### Study quality

The study quality is shown in Table 2. None of the studies had a low risk of bias on all quality domains, and only one study scored low on more than two domains. All studies had a high risk of bias on the domain of *study attrition,* and five studies had moderate to high risk of bias on the domain *risk factor measurements.* The domain that had the lowest risk of bias across the studies was *statistical analysis and reporting.* Disagreements between the two authors before consensus meeting were 18.8%, but the meeting resulted in agreement on all scores.

**Table 2:** Study quality assessment

Study	Study participation	Study attrition	Risk factor measurement	Outcome measurement	Confounding Measurement	Statistical analysis and reporting
**Dieck et al.** [37]	High	High	High	Moderate	High	High
**Grimby-Ekman et al.** [36]	Moderate	High	Moderate	Low	Moderate	Low
**Gustafsson et al.** [35]	High	High	Moderate	Moderate	High	Low
**Kanchanomai et al.** [34]	High	High	Low	Low	High	Moderate
**Siivola et al.** [39]	Low	High	Moderate	Low	Moderate	Low
**Timpka et al.** [38]	Low	High	High	High	Moderate	Low

**Study Participation:** Representativeness of the study sample; **Study Attrition:** Data from participants not lost to follow-up accurately represent the persons enrolled in the study;**Risk factor measurement:** The risk factor is measured in a similar, valid and reliable way for all participants; **Outcome measurement:** The outcome is measures in a similar, valid and reliable way for all participants; **Confounding measurement:** Important potential confounding factors are addressed; **Analysis:** The analysis is appropriate, and all primary outcomes are reported

### Risk factors for neck pain

A total of 56 potential risk factors were investigated in the six included studies, of which five were investigated in more than one study. The potential risk factors covered all ICF components in the model and a broad range of ICF domains; 1) body functions and structures (*n* = 24), 2) activities and participation (*n* = 15), 3) environmental (*n* = 10) and 4) personal factors (*n* = 7) (Table 3).

**Table 3:** Potential risk factors grouped into the international classification of function and disability (ICF)

Study	Body Functions and Structures	Activities and Participation	Environmental factors	Personal factors
**Dieck et al.** *From Table 6*^*a*^	• Shoulder elevation^c^ • Hip elevation^c^ • Deviation of the spine^c^ • Scoliosis: X^2^ = 0.001 (*p* = 0.98) • Kyphosis (normal): X^2^ = 6.38 (*p* = 0.09) • Lordosis (normal): X^2^ = 1.05 (*p* = 0.79) • Pelvic tilt (normal):X^2^ = 0.19 (*p* = 0.91)			
**Grimby Ekman et al.** [36] *From Table 6*^*a*^	• Asthma: crude OR = 2.0 (*p* = 0.046)^b^ • Asthma: adj. OR = 2.0 (0.996–3.91) • Perceived stress: adj. OR 1.7 (1.13–2.63)^b^ • Overweight: OR = 0.80 (*p* = 0.522)	• Computer use pattern: - One 4 h period without a break: adj. OR = 1.7 (0.941–2.94) - At least two 4 h periods without a break adj. OR = 1.8 (1.16–2.89)^b^ • Physical activity: OR = 0.99 (*p* = 0.599) • Breakfast regularly: OR = 0.81 (*p* = 0.423) • Work/study time: OR 1.0 (*p* = 0.889) • High work/study demands - Not affecting home life: adj. OR = 1.4 (0.913–2.20) - Affecting home life: adj. OR = 1.1 (0.596–2.12) • High home life demands: adj. OR = 2.2 (0.912–5.07) • Good relationships with superiors: OR = 0.67 (*p* = 0.280) • Good relationships with colleagues: adj. OR = 0.72 (0.354–1.48)		• Being female: adj. OR 3.1 (2.00–4.82)^b^ • Snuff use: OR = not reported • Smoking: OR = 1.2 (*p* = 0.685)
**Gustafsson et al.** [35] *From* table 3^a^		**One year follow-up:** - High stable, Short message service (SMS) (> 11 per day): adj. OR = 1.0 (0.69–1.58) - Stable SMS: OR = 1 **Five year follow up:** - High stable SMS: adj. OR = 1.3 (0.76–2.19) - Stable SMS: OR = 1		
**Kanchanomai et al.** [34] *From* Table 2 and 3^a^	• Neck extensor and flexor endurance^c^ • Neck range of motion (ROM), extension, flexion, rotation and lateral rotation ^c^ • Pectoralis major muscle length^c^ • Upper limb nerve tension test ^c^ • ROM elbow extension ^c^ • Chronic diseases^c^ • Mental health ^c^ • Body mass index^c^	• Elbows positioned at 90° angle while using computer - Yes: adj. OR = 1.00 - No: adj. OR = 1.52 (0.99–2.31) • Percent duration of mouse use while working at desk - < 70: adj. OR = 1.00 - ≥ 70: adj. OR = 0.66 (0.42–1.04) • Physical activity^c^ • Average amount of daily computer use^c^ • Years of computer use^c^ • Year of study - 1st year: adj. OR = 1.00 - 2nd year: adj. OR = 1.90 (1.08–3.35)^b^ - 3rd year: adj. OR = 0.96 (0.42–2.15) - 5th year: adj. OR = 7.09 (0.57–87.70) • Percent time of computer use for entertainment - < 70: adj. OR = 1.00 - ≥ 70: adj. OR = 0.44 (0.21–0.95)^b^	• Type of computer^c^ • Support of head, upper back, low back and arms while using computer • Computer screen positioned at eye level - Yes: adj. OR = 1.00 - No: adj. OR = 1.64 (1.13–2.36) ^b^ • Mouse height - Suitable: adj. OR = 1.00 - Too high: adj. 1.30 (0.82–2.10) - Too low: adj. OR = 0.52 (0.28–0.99)^b^ • Keyboard height - Suitable: adj. OR = 1.00 - Too low: adj. OR = 0.46 (0.17–1.20) - Too high: adj. OR = 2.18 (1.21–3.91)^b^	• Gender^c^ • Age^c^ • Field of study^c^
**Siivola et al.** [39] *From* table 3^a^	• Physical condition - Good or fairly good: RR = 1 - Moderate: RR = 1.3 (0.8–2.2) - Fairly poor or poor: RR = 0.9 (0.4–2.5) • Depressive mood - As continuous variable: RR = 1.1 (0.9–1.2) Symptoms decreased: RR = 1 (0.6–1.6) - Symptoms unchanged: RR = 1 - Symptoms increased: RR = 1.3 (0.8–2.1) • Psychosomatic symptoms score (crude): - As continuous variable: RR = 1 (1.0–1.1)* - Symptoms decreased: RR = 0.6 (0.3–1.0) - Symptoms unchanged: RR = 1 - Symptoms increased: RR = 1.5 (0.9–2.6) • BMI - Quartile 1: RR = 1 - Quartile 2: RR = 1 (0.5–1.9) - Quartile 3: RR = 1 (0.4–2.1) - Quartile 4: RR = 1 (0.5–1.9) • Height - Quartile 1: RR = 1 - Quartile 2: RR = 1.5 (0.7–3.1) - Quartile 3: RR = 1.6 (0.8–3.3) • Quartile 4: RR = 0.9 (0.4–2.1) • Panting and sweating in physical exercise (intensity of physical activity) - A lot: RR = 1 - Somewhat: RR = 1.5 (0.7–3.4) - A little/not at all/no exercise: RR = 1.3 (0.7–2.5)	• School achievement (grade points) - Quartile 1:RR = 1 - Quartile 2:RR = 1.7 (0.7–4.1) - Quartile 3: RR = 1 (0.4–2.3) - Quartile 4: RR = 1 (0.4–2.5) • Time spent on homework • School mark for physical education: RR = 0.9 (0.7–1.2) • Physical activity leisure time - Very active: RR = 1 - Fairly active: RR = 1.1 (0.6–2.2) - Fairly passive: RR = 1.4 (0.6–2.9) - Very passive: RR = 1.3 (0.6–2.8) • Type of leisure activity - Sport activities involving dynamic loading of upper extremities: RR = 0.8 (0.4–1.5) - Other sports: RR = 1.2 (0.3–4.7) - Hobbies that statically load the upper extremities: RR = 1.2 (0.3–4.7) - Other hobbies: RR = 1 (0.4–2.4)		• Gender - Male: 1 - Female: RR = 1.3 (0.8–2.2) • Seeking health care for NSP - Quartile 1: RR = 1 - Quartile 2: RR = 1.4 (0.7–2.9) - Quartile 3: RR = 1 (0.5–2.0) - Quartile 4:RR = 1.1 (0.6–2.3)
**Timpka et al.** [38] *From* table 2^a^	• Isometric muscle strength - Low: adj. RR = 0.93 (0.83–1.03) - High: adj. RR = 1.00 (0.90–1.10)			

**a** Results from tables in original studies

**b** Significant results

**c** Non-significant results, risk estimates not presented in original article

*OR* odds ratio, *adj. OR* adjusted odds ratio, *RR* relative risk, *adj. RR* adjusted relative risk, *p p*-value, *95% CI* 95% confidence interval, *SMS* short message service

### Risk factors investigated in more than one study

Five risk factors were investigated in more than one study: female sex, BMI, physical activity, duration of daily computer use and perceived stress. Three studies [34, 36, 39] investigated sex as a risk factor for neck pain with inconsistent results (Table 3). One study found female sex to be a risk factor [36], whereas the two other studies did not find any association [34, 39]. The overall quality of evidence was rated as very low as all the studies were considered phase 1 exploratory studies and were affected by serious study limitations, inconsistent results and imprecisions (Table 4).

**Table 4:** Overall quality of evidence table

Univariate Multivariate										GRADE factors							
Potential risk factors identified	Number of participants	Number of studies	+	0	–	+	0	–	Phase	Study limitations	Inconsistency	Indirectness	Imprecision	Publication bias	Moderate/ large effect size	Dose effect	Overall quality
Being female	895–954	3	1 2 0			1 0 0			1	x	x	√	x	√	x	NA	+
Body mass index	895–954	3	0 3 0			NA			1	x	√	√	x	√	x	x	+
Physical activity	895–954	3	0 3 0			NA			1	x	√	√	x	√	x	x	+
Mental stress	371–430	2	1 1 0			1 0 0			1	x	x	√	x	√	x	NA	+
Duration of computer use	791–850	2	1 1 0			1 1 0			1	x	x	√	x	√	x	x	+
Not having computer screen at eye level	524	1	NA			1 0 0			1	x	NA	√	√	NA	x	NA	+
Keyboard positioned too high	524	1	NA			1 0 0			1	x	NA	√	x	NA	√	x	+
Being a 2nd year student	524	1	NA			1 0 0			1	x	NA	√	x	NA	x	x	+
Computer mouse height too low	524	1	NA			0 0 1			1	x	NA	√	x	NA	x	x	+
Using computer for entertainment ≥70% of time	524	1	NA			0 0 1			1	x	NA	√	x	NA	x	NA	+

For uni- and multivariate analyses: + represents number of significant effects with a positive OR; 0 represents number of non-significant effects; − represents number of significant effects with a negative OR. Phase means phase of investigation. For GRADE factors: ✓, no serious limitations; ✕, serious limitations (or not present for moderate/large effect size, dose effect) NA, unable to rate item based on available information. For overall quality of evidence: + very low; ++ low; +++ moderate; ++++ high. Only factors investigated in more than one study or which showed statistical significance in one study are included in the table. Number of participants vary because of inaccurate reporting of participants in one of the studies

High BMI was investigated in three studies [34, 36, 39], and none of them showed any association between BMI and neck pain (Table 3). The studies had very low-quality evidence, as they were all considered phase 1 exploratory studies affected by serious study limitations and imprecisions (Table 4).

Self-reported physical activity level was investigated in three low-quality studies [34, 36, 39], and operationalised differently across these studies: exercise or training the last 7 days, weekly physical activity level, and type and intensity of physical activity in leisure time. None of these studies showed an association between physical activity and neck pain (Table 3). All of the studies were considered to be phase 1 exploratory studies with serious study limitations and imprecision (Table 4).

Duration of daily computer use was measured in two studies [34, 36] with inconsistent results (Table 3). Using a computer for at least two 4 h periods without a break was associated with neck pain [34], whereas computer use for neither less nor more than 3 h per day was not associated with neck pain [36]. The overall study quality was rated as very low as they were exploratory studies, and had serious study limitations, inconsistent results and imprecisions (Table 4).

Perceived stress was investigated in two studies [36, 39], also operationalised in different ways across the studies (Table 3). Grimby-Ekman et al. reported that perceived stress was a risk factor for neck pain [36], both after one and 2 years. Siivola et al. found a weak association between neck pain and mental stress in unadjusted analysis in males, but not in females [39]. Because the evidence of these factors came from phase 1 exploratory studies and was influenced by severe study limitations, inconsistency and imprecision, the overall quality of the evidence was rated as very low (Table 4).

### Significant risk factors investigated in only one study

Of the risk factors included in this review, only three risk factors and two protective factors were significantly associated with neck pain. All these factors were identified from one study. Kanchanomai et al. [34] reported not having computer screen at eye level as a risk factor for developing neck pain and having the mouse positioned too low as a protective factor compared to having the mouse positioned at a ‘suitable’ height (Table 3). The same study found an association between persistent neck pain and having the keyboard positioned too high compared to ‘suitable’, and that 2nd year students were at significantly higher risk of experiencing persistent neck pain compared to 1st year students. Using a computer for entertainment ≥70% of the time was also found to be a protective factor for persistent neck pain. The overall quality of these risk and protective factors was rated as very low because all were investigated in a single exploratory study, which was affected by serious study limitations (Table 4).

## Discussion

This systematic review identified 56 potential risk factors for their impact on neck pain in the age group of 18–29 years. The risk factors were investigated in six prospective cohort studies and represented constructs from a wide range of domains according to the ICF framework of health and functioning. Only five of the 56 potential risk factors were investigated in more than one study: female sex, BMI, physical activity, duration of daily computer use and perceived stress. Inconsistent results were found for female sex, duration of daily computer use and perceived stress, whereas BMI and physical activity level showed no association with neck pain. All the potential risk factors were identified from low-quality evidence. Large heterogeneity and the small number of studies made it inappropriate to conduct a meta-analysis.

BMI and physical activity level were not associated with neck pain according to three studies in this review. This is in line with findings in a previous systematic review by Huguet et al., which investigated risk factors for musculoskeletal pain in children and adolescents aged 5–18 years [40]. In adults, however, one systematic review found high BMI (> 30 kg/m^2^) to be a strong risk factor for neck pain [20]. Inconsistent results across reviews might be due to differences in age groups, but also the complexity involved in investigating physical activity and BMI. For example, there might be a u-shaped curve with more pain in those with inactivity and excessive activity, compared to those with moderate activity [41]. Similarly, high BMI might be due to either a high level of body fat or muscle mass, which might influence pain in different ways. These issues were not investigated in any of the included studies or in previous reviews of risk factors.

Female sex demonstrated inconsistent results across two studies in this review, which is in line with the previous review of children and adolescents [40], as well as two other reviews on adults [20, 21]. Inconsistent findings were also found across two studies for the impact of perceived stress on neck pain. Only one study in our review investigated depression as a risk factor for neck pain [39] and found no association. This finding is contrary to what has been reported in studies among children, adolescents and adults [17, 20]. There is little knowledge on how stress and other mental factors might impact neck pain in young adults.

Most of the investigated risk factors showed no association with neck pain, and most of the studies did not add any explanations for their non-significant results. One study [34] did not report risk estimates of the non-significant results, which makes the direction of the results unclear. Different study populations hindered directly comparison of the results from this review to previous studies of adolescents and adults, which may explain some of the inconsistencies in the results. As all the identified risk factors in this review are based on few studies with low-quality evidence, the findings might be spurious and need to be investigated in large studies with high quality.

The classification of risk factors according to the ICF framework demonstrated that most research until now covered risk factors within daily activities and physical body functions, whereas few potential personal and environmental risk factors have been investigated. Importantly, factors specifically relevant to the life phase of young adulthood, such as educational level and digital media, have been less studied.

All the risk factors identified in the present study showed a very low level of evidence according to the GRADE framework. The included studies had mainly exploratory designs, with serious study limitations, e.g. imprecision due to high loss to follow-up, and small sample sizes. Few studies reported if neck pain and risk factors were measured with valid and reliable measurements. Neck pain was not defined similarly regarding frequency, localisation, duration or intensity. The measurement assessments for the same risk factors also varied across studies, which precluded comparison and might explain inconsistent results. The high risk of bias among these studies should be considered when interpreting the results.

### Strengths

To our knowledge, this is the first systematic review to summarise the evidence of risk factors for neck pain in young adulthood. We used a broad search strategy, covering various databases, minimising the chance of missing relevant articles. The inclusion of prospective cohort studies only increased the chance of finding the temporal sequence between risk factors and neck pain, and avoided recall bias compared to retrospective studies. The sorting of concepts and constructs according to the ICF framework provided an overview of which type of constructs and domains that have been explored until now.

### Limitations

One limitation of this study is that we did not have any time-oriented requirements of the duration of neck pain. Consequently, we might have included studies with populations with both new episodes as well as recurrent neck pain. The reason for including studies with participants that might have had a previous episode of neck pain was the known difficulty of identifying a true pain-free population. Recall bias disturbs accuracy when asking participants if they previously have experienced an episode of neck pain, which makes identifying the first ever-episode of neck pain a challenging task [42]. Further, by including studies that measured either the risk factor nor the outcome in the age 18–29 years, we ended up including two studies measuring risk factors in adolescents (15 and 17 years of age) [38, 39]. This led to slightly younger study samples that we targeted, and did not necessarily, investigate risk factors specifically relevant for the period of young adulthood. In addition, heterogeneity between studies such as lack of standardised measurements either of neck pain, risk factors or follow-up period made it difficult to combine the findings in a meta-analysis, which would have provided more accurate results of risk factors for neck pain.

### Implications

The findings from this systematic review indicate that there is a strong need for more high-quality prospective cohort studies of neck pain in this important life phase in order to elucidate the development of neck pain. Future studies should preferably include individuals who are pain-free at baseline or who never have experienced a previous episode of neck pain in order to be able to assess the potential impact of risk factors on the development of neck pain. Standardised methods should be used in order to enhance comparison of results as well as merging data sets across studies in meta-analysis. Importantly, future studies need to cover other types of potential important risk factors, reflecting current trends and aspects of young adults. Factors such as risk behaviour [8], digital habits [43], resilience [44], food insecurity [45] friendship [46], and loneliness [47], are all factors suggested to be relevant in young adulthood and might be important factors to investigate further in relationship with neck pain. More knowledge on significant risk factors for the development of recurrent and chronic neck pain in young adulthood will form the basis for developing effective prevention initiatives. Especially, since young adulthood is a period of life when implementation strategies for changes may be effective, and personal, professional and health trajectories are shaped and established [48, 49].

## Conclusion

This systematic review, including 56 potential risk factors investigated in six prospective studies, did not identify confident evidence for any distinct risk factors for neck pain in young adults. The main finding is the paucity of high-quality prospective studies investigating risk factors for neck pain from a pain-free state in this age group. The high number of low-quality studies investigating a large amount of potential risk factors for neck pain with only spurious findings is alarming. Therefore, there is a strong need for more high-quality prospective studies in this field, in which the study population and outcome is clearly defined, and where the rationale for each of the selected potential risk factors is provided.

## Supplementary information

**Additional file 1:** The PRISMA checklist.

**Additional file 2:** Search strategies. Search strategies for AMED, Cinahl, EMBASE, MEDLINE, PsychINFO, SportsDiscus and Web of Science.

**Additional file 3:** Excluded studies. Studies identified in the full-text search with reasons for exclusion.

## Data Availability

All data generated or analysed during this study are included in this published article (supplementary file 3).

## Abbreviations

BMI: Body mass index
GBD: Global Burden of Disease Study
GRADE: Grading of Recommendations Assessments, Development and Evaluation
CI: Confidence interval
ICF: International Classification of Functioning, Disability and Health
PRISMA: Preferred Reporting Items for Systematic Reviews and Meta-Analyses
QUIPS: Quality in Prognosis Studies assessment tool
YLD: Years lived with disabilities
