# Supplementary material for: Risk factors for non-specific neck pain in young adults. A systematic review
Source: BMC Musculoskelet Disord. 2020 Jun 9;21:366. doi: 10.1186/s12891-020-03379-y (PMC7285427; doi:10.1186/s12891-020-03379-y)
Supplement: Supplementary file 2 — Additional file 2: Search strategies. Search strategies for AMED, Cinahl, EMBASE, MEDLINE, PsychINFO, SportsDiscus and Web of Science. [file 12891_2020_3379_MOESM2_ESM.docx]

**Appendix 2. Search strategies**

**Database:** AMED (Allied and Complementary Medicine) 1985 to January 2019, via Ovid

**Date:** 10.09.2019

**Results:** 22

| **1.** exp Adolescent/ |
| --- |
| **2.** exp Students/ |
| **3.** ((young* or prime* or emerging* or early) adj2 (people or person* or adult*)).ti,ab. |
| **4.** (adolescen* or juvenile or youth*).ti,ab. |
| **5.** 1 or 2 or 3 or 4 |
| **6.** exp risk factors/ |
| **7.** exp Risk/ |
| **8.** exp Probability/ |
| **9.** exp Prognosis/ |
| **10.** (prognos* or predict* or indicate* or cause*).ti,ab. |
| **11.** (risk adj2 (genetic or behavio?r* or patient* or population* or recurrence* or attributable)).ti,ab. |
| **12.** 6 or 7 or 8 or 9 or 10 or 11 |
| **13.** exp Neck pain/ |
| **14.** exp Musculoskeletal Pain/ |
| **15.** ((neck or cervical or musculoskeletal) adj2 (pain or ache* or complain* or problem*)).ti,ab. |
| **16.** 13 or 14 or 15 |
| **17.** exp prospective studies/ or exp cohort studies/ |
| **18.** exp Follow up studies/ or exp Etiology/ |
| **19.** exp Epidemiology/ |
| **20.** exp longitudinal studies/ |
| **21.** ((longitudinal or observational or follow-up) adj2 stud*).ti,ab. |
| **22.** (cohort* or epidemiologic* or prospective*).ti,ab. |
| **23.** 17 or 18 or 19 or 20 or 21 or 22 |
| **24.** 5 and 12 and 16 and 23 |
| **25.** limit 24 to (danish or english or norwegian or swedish) |

**Database:** Cinahl via Ebscohost

**Date:** 10.09.2019

**Results:** 404

| **S1** (MH "Young Adult") |
| --- |
| **S2** (MH "Adolescence+") |
| **S3** (MH "Students+") |
| **S4** TI ( ((young* or prime* or emerging* or early) N2 (people or person* or adult*)) ) OR AB ( ((young* or prime* or emerging* or early) N2 (people or person* or adult*)) ) |
| **S5** TI ( adolescen* or juvenile* or youth ) or AB ( adolescen* or juvenile* or youth ) |
| **S6** TI ( ((high school or middle school or college or university or dropout*) N2 stud*)) ) OR AB ( ((high school or middle school or college or university or dropout*) N2 stud*)) ) |
| **S7** S1 OR S2 OR S3 OR S4 OR S5 OR S6 |
| **S8** (MH "Risk Factors+") |
| **S9** (MH "Risk Assessment") |
| **S10** (MH "Probability+") |
| **S11** (MH "Prognosis+") |
| **S12** TI ( ( (risk N2 (factor* or assessment*)) ) OR AB ( ( (risk N2 (factor* or assessment*)) |
| **S13** TI ( (prognos* or predict* or indicate* or cause*) ) OR AB ( (prognos* or predict* or indicate* or cause*) ) |
| **S14** TI ( ((predict* or association* or predispos*) N2 factor*) ) OR AB ( ((predict* or association* or predispos*) N2 factor*) ) |
| **S15** TI ( (risk N2 (genetic or behavio?r* or patient* or population* or recurrence* or attributable)) ) OR AB ( (risk N2 (genetic or behavio?r* or patient* or population* or recurrence* or attributable)) |
| **S16** S8 OR S9 OR S10 OR S11 OR S12 OR S13 OR S14 OR S15 |
| **S17** (MH "Neck Pain") |
| **S18** (MH "Muscle Pain") |
| **S19** TI ( ((neck or cervical or musculoskeletal) N2 (pain or ache* or complain* or problem*)) ) OR AB ( ((neck or cervical or musculoskeletal) N2 (pain or ache* or complain* or problem*)) |
| **S20** S17 OR S18 OR S19 |
| **S21** (MH "Prospective Studies+") |
| **S22** (MH "Epidemiological Research+") |
| **S23** TI ( ((longitudinal or observational or follow-up) N2 stud*) ) OR AB (((longitudinal or observational or follow-up) N2 stud*) ) |
| **S24** TI ( (cohort* or epidemiologic* or prospective*) ) OR AB ( (cohort* or epidemiologic* or prospective*) ) |
| **S25** S21 OR S22 OR S23 OR S24 |
| **S26** S7 AND S16 AND S20 AND S25 |
| **S27** S7 AND S16 AND S20 AND S25 |
| **S28** Limiters - Human; Language: Danish, English, Norwegian, Swedish |
| **S29** S27 AND S28 |

**Database:** Embase, 1974 to January 2019 via Ovid

**Date:** 10.09.19

**Results:** 4216

| **1.** exp young adult/ |
| --- |
| **2.** adolescent/ |
| **3.** exp student/ |
| **4.** exp school dropout/ |
| **5.** ((young* or prime* or emerging* or early) adj2 (people or person* or adult*)).kw,tw. |
| **6.** (adolescen* or juvenile or youth*).kw,tw. |
| **7.** ((high school or middle school or college or university or dropout*) adj2 stud*).kw,tw. |
| **8.** 1 or 2 or 3 or 4 or 5 or 6 or 7 |
| **9.** exp risk factor/ |
| **10.** exp risk/ |
| **11.** exp risk assessment/ |
| **12.** exp probability/ |
| **13.** exp prognosis/ |
| **14.** (risk adj2 (factor* or assessment*)).kw,tw. |
| **15.** (prognose* or predict* or indicate* or cause*).kw,tw. |
| **16.** ((predict* or association* or predispos*) adj2 factor*).kw,tw. |
| **17.** (risk adj2 (genetic or behavio?r* or patient* or population* or recurrence* or attributable)).kw,tw. |
| **18.** 9 or 10 or 11 or 12 or 13 or 14 or 15 or 16 or 17 |
| **19.** exp neck pain/ |
| **20.** exp musculoskeletal pain/ or exp myalgia/ |
| **21.** ((neck or cervical or musculoskeletal) adj2 (pain or ache* or complain* or problem*)).kw,tw. |
| **22.** 19 or 20 or 21 |
| **23.** exp prospective study/ or exp cohort analysis/ or exp follow up/ |
| **24.** exp epidemiology/ |
| **25.** exp longitudinal study/ |
| **26.** exp observational study/ |
| **27.** ((longitudinal or observational or follow-up) adj2 stud*).kw,tw. |
| **28.** (cohort* or epidemiologic* or prospective*).kw,tw. |
| **29.** 23 or 24 or 25 or 26 or 27 or 28 |
| **30.** 8 and 18 and 22 and 29 |
| **31.** limit 30 to (human and (danish or english or norwegian or swedish)) |

**Database:** Ovid MEDLINE (R) and Epub Ahead of Print, In-Process & Other Non-Indexed Citations and Daily 1946 to January 2019

**Date:** 10.09.2019

**Results:** 802

| **1.** exp young adult/ |
| --- |
| **2.** adolescent/ |
| **3.** exp student/ |
| **4.** exp school dropout/ |
| **5.** ((young* or prime* or emerging* or early) adj2 (people or person* or adult*)).kw,tw. |
| **6.** (adolescen* or juvenile or youth*).kw,tw. |
| **7.** ((high school or middle school or college or university or dropout*) adj2 stud*).kw,tw. |
| **8.** 1 or 2 or 3 or 4 or 5 or 6 or 7 |
| **9.** exp risk factor/ |
| **10.** exp risk/ |
| **11.** exp risk assessment/ |
| **12.** exp probability/ |
| **13.** prognosis/ |
| **14.** (risk adj2 (factor* or assessment*)).kw,tw. |
| **15.** (prognose* or predict* or indicate* or cause* or ((predict* or association* or predispos*) adj2 factor*)).kw,tw. |
| **16.** (risk adj2 (genetic or behavio?r* or patient* or population* or recurrence* or attributable)).kw,tw. |
| **17.** 9 or 10 or 11 or 12 or 13 or 14 or 15 or 16 |
| **18.** exp neck pain/ |
| **19.** exp musculoskeletal pain/ or exp myalgia/ |
| **20.** ((neck or cervical or musculoskeletal) adj2 (pain or ache* or complain* or problem*)).kw,tw. |
| **21.** 18 or 19 or 20 |
| **22.** exp cohort analysis/ |
| **23.** exp epidemiology/ |
| **24.** exp longitudinal study/ |
| **25.** exp prospective study/ |
| **26.** exp observational study/ |
| **27.** exp follow up/ |
| **28.** ((Cohort or epidemiologic* or prospective or (longitudinal or observational or follow-up)) adj2 stud*).kw,tw. |
| **29.** 22 or 23 or 24 or 25 or 26 or 27 or 28 |
| **30.** 8 and 17 and 21 and 29 |

**Database:** Ovid PsychINFO 1806 to January 2019, via Ovid

**Date:** 10.09.2019

**Results:** 46

| **1.** exp Emerging Adulthood/ |
| --- |
| **2.** exp STUDENTS/ |
| **3.** ((young* or prime* or emerging* or early) adj2 (people or person* or adult*)).tw. |
| **4.** (adolescen* or juvenile or youth*).tw. |
| **5.** ((high school or middle school or college or university or dropout*) adj2 stud*).tw. |
| **6.** 1 or 2 or 3 or 4 or 5 or 6 |
| **7.** exp Risk Factors/ |
| **8.** exp RISK ASSESSMENT/ |
| **9.** exp PROBABILITY/ |
| **10.** exp PROGNOSIS/ |
| **11.** (risk adj2 (factor* or assessment*)).tw. |
| **12.** (prognos* or predict* or indicate* or cause*).tw. |
| **13.** ((predict* or association* or predispos*) adj2 factor*).tw. |
| **14.** (risk adj2 (genetic or behavio?r* or patient* or population* or recurrence* or attributable)).tw. |
| **15.** 8 or 9 or 10 or 11 or 12 or 13 or 14 or 15 |
| **16.** ((neck or cervical or musculoskeletal) adj2 (pain or ache* or complain* or problem*)).tw. |
| **17.** exp Prospective Studies/ |
| **18.** exp Cohort Analysis/ |
| **19.** exp Followup Studies/ |
| **20.** exp EPIDEMIOLOGY/ |
| **21.** exp Longitudinal Studies/ |
| **22.** ((longitudinal or observational or follow-up) adj2 stud*).tw. |
| **23.** (cohort* or epidemiologic* or prospective*).tw. |
| **24.** 19 or 20 or 21 or 22 or 23 or 24 |
| **25.** 7 and 16 and 17 and 25 |
| **26.** limit 26 to (human and (danish or english or norwegian or swedish)) |

**Database:** SportsDiscus via Ebscohost

**Date:** 10.09.2019

**Results:** 36

| **S1** DE "YOUNG adults" |
| --- |
| **S2** DE "TEENAGERS" |
| **S3** DE "STUDENTS" |
| **S4** TI ( (young* or prime* or emerging* or early) N2 (people or person* or adult*) ) OR AB ( (young* or prime* or emerging* or early) N2 (people or person* or adult*) ) |
| **S5** TI ( adolescen* or juvenile or youth ) OR AB ( adolescen* or juvenile or youth ) |
| **S6** TI ( (high school or middle school or college or university or dropout*) N2 stud* ) OR AB ( (high school or middle school or college or university or dropout*) N2 stud* ) |
| **S7** S1 OR S2 OR S3 OR S4 OR S5 OR S6 |
| **S8** DE "DISEASE risk factors |
| **S9** DE "HEALTH risk assessment" |
| **S10** TI ( (risk N2 (factor* or assessment*) ) OR AB ( (risk N2 (factor* or assessment*) ) |
| **S11** TI ( (prognos* or predict* or indicate* or cause*) ) OR AB ( (prognos* or predict* or indicate* or cause*) |
| **S12** TI ( (predict* or association* or predispos*) ) OR AB ( (predict* or association* or predispos*) ) |
| **S13** TI ( risk N2 (genetic or behavio?r* or patient* or population* or recurrence* or attributable) ) OR AB ( risk N2 (genetic or behavio?r* or patient* or population* or recurrence* or attributable) ) |
| **S14** S8 OR S9 OR S10 OR S11 OR S12 OR S13 |
| **S15** DE "NECK pain" |
| **S16** DE "MYALGIA" |
| **S17** TI ( ((neck or cervical or musculoskeletal) N2 (pain or ache* or complain* or problem*)) ) OR AB ( ((neck or cervical or musculoskeletal) N2 (pain or ache* or complain* or problem*)) ) |
| **S18** S15 OR S16 OR S17 |
| **S19** DE "COHORT analysis" |
| **S20** DE "EPIDEMIOLOGY" |
| **S21** TI ( ((longitudinal or observational or follow-up) N2 stud*) ) ) OR AB ( ((longitudinal or observational or follow-up) N2 stud*) ) ) |
| **S22** TI ( (cohort* or epidemiologic* or prospective*) ) OR AB ( (cohort* or epidemiologic* or prospective*) ) |
| **S23** S19 OR S20 OR S21 OR S22 |
| **S24** S7 AND S14 AND S18 AND S23 |
| **S25** S7 AND S14 AND S18 AND S23 |

**Database:** Web of Science 1987 to January 2019

**Date:** 10.09.2019

**Results:** 287

| **#1 TOPIC:** ((((young* OR prime* OR emerging* OR early) NEAR/2 (people OR person* OR adult*))))  *DocType=All document types; Language=All languages;* |
| --- |
| **#2 TOPIC:** (((adolescen* OR juvenile* OR youth)))  *DocType=All document types; Language=All languages* |
| **#3** TS=(high school student* or middle school student* or college student* or university student*or student dropout*)  *DocType=All document types; Language=All languages;* |
| **#4** #3 OR #2 OR #1  *DocType=All document types; Language=All languages;* |
| **#5 TOPIC:** (((risk NEAR/2 (factor* OR assessment*))))  *DocType=All document types; Language=All languages;* |
| **#6 TOPIC:** (((prognos* OR predict* OR indicate* OR cause*)))  *DocType=All document types; Language=All languages;* |
| **#7 TOPIC:** (((predict* OR association* OR predispos*) NEAR/2 factor*))  *DocType=All document types; Language=All languages;* |
| **#8 TOPIC:** ((risk NEAR/2 (genetic OR behavio?r* OR patient* OR population* OR recurrence* OR attributable)))  *DocType=All document types; Language=All languages;* |
| **#9** #8 OR #7 OR #6 OR #5  *DocType=All document types; Language=All languages;* |
| **#10 TOPIC:** ((((neck OR cervical OR musculoskeletal) NEAR/2 (pain OR ache* OR complain* OR problem*))))  *DocType=All document types; Language=All languages;* |
| **#11 TOPIC:** (((longitudinal OR observational OR follow-up) NEAR/2 stud*))  *DocType=All document types; Language=All languages;* |
| **#12 TOPIC:** ((cohort* OR epidemiologic* OR prospective*))  *DocType=All document types; Language=All languages;* |
| **#13 #12 OR #11**  **DocType=All document types; Language=All languages;** |
| **#14** (#13 AND #10 AND #9 AND #4) *AND* **LANGUAGE:** (English OR Danish OR Norwegian OR Swedish)  *DocType=All document types; Language=All languages;* |
