# Supplementary material for: Risk factors for non-specific neck pain in young adults. A systematic review
Source: BMC Musculoskelet Disord. 2020 Jun 9;21:366. doi: 10.1186/s12891-020-03379-y (PMC7285427; doi:10.1186/s12891-020-03379-y)
Supplement: Supplementary file 3 — Additional file 3: Excluded studies. Studies identified in the full-text search with reasons for exclusion. [file 12891_2020_3379_MOESM3_ESM.docx]

**Appendix 3. Studies identified in the full-text search with reasons for exclusion**

| **Age group** | **Combined outcome** | **Study design** | **Conference abstracts** | **Study population** | **Not relevant outcome** |
| --- | --- | --- | --- | --- | --- |
| Marklund et al. (2010) | Hellsing et al. (2000) | Sorour et al. (2012) | Alvarado (2018) | Auvinen et al. (2010) | Kanchanomai et al.(2013) |
| Løvgren et al. (2014) | Dick et al. (2015) | Shittu et al. (2016) | Barrense-Dias et al. (2018) | Clark et al. (2016) | Leino-Arjas et al. (2018) |
| Widanarko et al. (2015) | Kroner-Herwig et al. (2017) | Yeun et al. (2017) | Bunn et al. (2017) | Hanvold et al.(2014) | Myrtveit et al. (2013) |
| Sadeghian et al. (2013) | Solodaki et al. (2013) | Abaraogu et al. (2018) | Grimby-Ekman et al. (2011) | Hanvold et al.(2013) | Wenzel et al. (2012) |
| Barnekow-Bergkvist et al. (1998) | Stanford et al. (2007) | Al-Sheri et al. (2018) |  | Hertzberg et al. (1985) | Brattberg et al. (2004) |
| Grossi et al. (2009) | Arvidsson et al. (2008) | De Noroes Ramos et al. (2018) |  | Laimi et al. (2014) | Lourenco et al. (2017) |
| Kääriä et al. (2009) | Ballenberger et al. (2018) | Kedra et al. (2017) |  |  |  |
| Sitthipornvorakul et al. (2015) | Boström et al. (2008) | Noll et al. (2016) |  |  |  |
| Ståhl et al. (2008) | Brady et al. (2016) | Lee et al. (2014) |  |  |  |
| Aasa et al. (2016) | Generaal et al.(2017) | Noll et al. (2019) |  |  |  |
| Bugjska et al. (2013) | Gonzalez et al. (2012) | Schmaling and Nounou (2019) |  |  |  |
| Ehrmann Feldmann et al. (2002) | Kvalheim et al. (2013) |  |  |  |  |
| Hartvigsen et al. (2009) | Madsen et al. (2018) |  |  |  |  |
| Hill et al. (2004) | Paananen et al. (2015) |  |  |  |  |
| Holth et al. (2008) | Christensen et al. (2014) |  |  |  |  |
| Iversen et al. (2015) |  |  |  |  |  |
| Krause et al. (2010) |  |  |  |  |  |
| Lindegard et al. (2012) |  |  |  |  |  |
| Mikkelson et al. (2006) |  |  |  |  |  |
| Nakamura et al. (2014) |  |  |  |  |  |
| Nilsen et al. (2011) |  |  |  |  |  |
| Nolet et al. (2011) |  |  |  |  |  |
| Nolet et al (2012) |  |  |  |  |  |
| Nyman et al. (2009) |  |  |  |  |  |
| Paanalahti et al. (2014) |  |  |  |  |  |
| Paksaichol et al. (2014) |  |  |  |  |  |
| Sarquis et al. (2016) |  |  |  |  |  |
| Sihawong et al. (2012) |  |  |  |  |  |
| Viikari-Juntura et al. (1991) |  |  |  |  |  |
| Andersen et al. (2003) |  |  |  |  |  |
| Andersen et al. (2013) |  |  |  |  |  |
| Christensen et al. (2014) |  |  |  |  |  |
| Croft et al. (2001) |  |  |  |  |  |
| Laurèn et al. (1997) |  |  |  |  |  |
| Paanalahti et al. (2014) |  |  |  |  |  |
| Palmlöf et al. (2016) |  |  |  |  |  |
| Palmlöf et al. (2012) |  |  |  |  |  |
| Posch et al. (2019) |  |  |  |  |  |
| Rugulies et al. (2008) |  |  |  |  |  |
| Shahidi et al. (2015) |  |  |  |  |  |
| Yang et al. (2014) |  |  |  |  |  |
| Ariens et al. (2001) |  |  |  |  |  |
| Luime et al. (2004) |  |  |  |  |  |

**Age group:** studies that did not include separate analysis for young adults (18-29 years); **Combined outcome:** studies that combine neck pain with other musculoskeletal disorders; **Study design:** not prospective cohort or registry studies; **Conference abstracts:** only conference abstract existed; **Study population:** included participants with pain at baseline; **Not relevant outcome;** the studies have not measured neck pain as an outcome.
